# Association between pain intensity and body composition in adults with chronic non‐specific low back pain: A systematic review and meta‐analysis

**DOI:** 10.1111/obr.13875

**Published:** 2024-12-02

**Authors:** Melanie Liechti, Massimo Menegon, Alexander Philipp Schurz, Jan Taeymans, Heiner Baur, Ron Clijsen, Anneleen Malfliet, Nathanael Lutz

**Affiliations:** ^1^ School of Health Professions Bern University of Applied Sciences Bern Switzerland; ^2^ Department of Movement and Sport Sciences, Faculty of Physical Education and Physiotherapy Vrije Universiteit Brussel Brussels Belgium; ^3^ Pain in Motion Research Group (PAIN), Department of Physiotherapy, Human Physiology and Anatomy, Faculty of Physical Education and Physiotherapy Vrije Universiteit Brussel Brussels Belgium; ^4^ Faculty of Medicine University of Bern Bern Switzerland; ^5^ Rehabilitation and Exercise Science Laboratory (RESLab), Department of Business Economics, Health, and Social Care University of Applied Sciences and Arts of Southern Switzerland Landquart/Manno Switzerland; ^6^ International University of Applied Sciences THIM Landquart Switzerland; ^7^ Research Foundation – Flanders (FWO) Brussels Belgium; ^8^ Chronic Pain Rehabilitation, Department of Physical Medicine and Physiotherapy University Hospital Brussels Brussels Belgium; ^9^ Pain in Motion International Research Consortium Brussels Belgium

**Keywords:** back pain, body composition, overweight, pain intensity

## Abstract

**Introduction:**

This systematic review and meta‐analysis evaluated the association between pain intensity and different body composition measures in adults suffering from chronic non‐specific low back pain (CNLBP).

**Methods:**

A systematic literature search across five databases—PubMed, Embase, CINAHL, Web of Science, and the Cochrane Library—was conducted. It identified observational studies published until January 2024. A meta‐analysis was conducted incorporating a random‐effects approach with Fisher's Z transformation. A critical appraisal of the included studies' quality was conducted.

**Results:**

Twenty‐two studies were included. Of those, 20 were meta‐analyzed, revealing positive, very weak correlations between pain intensity and body mass index (*r* = 0.11; 95% CI: 0.04 to 0.18), waist–hip ratio (*r* = 0.10; 95% CI: −0.14 to 0.34) and waist circumference (*r* = 0.09; 95% CI: −0.28 to 0.44). Not pooled study findings (e.g., hip circumference and body fat percentage) were narratively summarized, revealing 13 positive and four negative associations between pain intensity and body composition measures. Studies showed a substantial risk of bias due to unadjusted confounding factors and limited transferability of findings.

**Conclusion:**

This systematic review and meta‐analysis provided very low‐quality evidence for a positive, very weak association between pain intensity and body composition measures in adults with overweight and obesity suffering from CNLBP. The included studies had a substantial risk of bias due to their observational and cross‐sectional study designs, which prevents recommendations for clinical practice. Randomized controlled trials are needed to investigate the causal effect of interventions on body composition measures and pain intensity.

**Abbreviations**
BIA: bioimpedance analysis
BMI: body mass index
CASP: Critical Appraisal Skills Programme
CI: confidence interval
CLBP: chronic low back pain
CMDQ: Cornell Musculoskeletal Discomfort Questionnaire
CNLBP: chronic non‐specific low back pain
CPGQ: Chronic Pain Grade Questionnaire
DALY: disability‐adjusted life year
DXA: dual‐energy X‐ray absorptiometry
HC: hip circumference
INPLASY: International Platform of Registered Systematic Review and Meta‐analysis Protocols
LBP: low back pain
MeSH: Medical Subject Headings
NRS: Numeric (Pain) Rating Scale
OR: odds ratio
PRISMA: Preferred Reporting Items for Systematic Reviews and Meta‐Analyses
SDS: Semantic Differential Scale
TBA: total body adiposity
TBFM: total body fat mass
TBW: total body water
VAS: Visual Analog Scale
WC: waist circumference
WHO: World Health Organization
WHR: waist–hip ratio

## 1 INTRODUCTION

In Europe, almost 60% of adults were affected by overweight or obesity in 2022.^1^ Living with overweight or obesity increases the risk of comorbidities like cardiovascular diseases, diabetes mellitus, cancer, or musculoskeletal disorders.^2^, ^3^, ^4^ Further, it is a leading risk factor for disability and is linked to increased mortality.^1^, ^5^ These wide‐reaching health impacts are associated with financial consequences. In 2019, $60.5 billion in healthcare costs and $120.2 billion in productivity losses due to morbidity were estimated for musculoskeletal disorders caused by overweight or obesity.^6^

There is growing evidence that overweight and obesity could be influential factors in low back pain (LBP) conditions. Prevalence of chronic LBP (CLBP) is higher among people with overweight and obesity as compared to individuals with normal weight, as overweight or obesity has been identified as a predictor of CLBP with odds ratios (ORs) ranging from 1.33 to 3.7.^7^, ^8^ The definition of CLBP is a controversial topic, as “low back pain” describes a symptom and is used differently among studies.^9^ In this systematic review and meta‐analysis, chronic non‐specific LBP (CNLBP) is defined as LBP that persists or fluctuates for at least 3 months, with no identifiable underlying pathological cause.^10^, ^11^ Studies using the term “CLBP,” which may refer to both, specific and non‐specific LBP conditions, were included in the systematic review because specific causes of LBP are identified in a small minority of cases only.^9^, ^10^

LBP is known to be one of the most important drivers of the largest increase in disability‐adjusted life years (DALYs) worldwide between 1990 and 2019 across all age groups (range 0 to ≥75 years).^12^ Several reviews acknowledged the association between LBP and different measures of overweight or obesity, including body mass index (BMI), body fat mass, waist–hip ratio (WHR), or waist circumference (WC).^8^, ^13^, ^14^, ^15^, ^16^ Various theories of the interaction between LBP and overweight or obesity in humans have been proposed. For example, obesity may increase mechanical loading of the spine^17^, ^18^ or adipose tissue, acting as an endocrine organ, releases pro‐inflammatory markers, which might induce pain.^13^, ^19^, ^20^, ^21^ Hence, overweight and obesity itself could be a contributing factor to increased nociception in people suffering from CNLBP.

On the other hand, whether pain intensity itself is related to overweight and obesity in adults with CNLBP remains unclear. A precise measurement of pain intensity and body composition in people with CNLBP and overweight or obesity could improve the understanding of potential mechanisms linking pain with overweight and obesity. Because BMI is a measure of adiposity but does not account for body fat distribution,^22^, ^23^ other measures of body composition such as (i) the percentage of body fat assessed by bioimpedance analysis (BIA) or dual‐energy X‐ray absorptiometry (DXA), (ii) anthropometric measures, such as WHR and WC, and (iii) subcutaneous adipose tissue thickness assessed by ultrasound or skinfold caliper should be investigated.

Therefore, this systematic review and meta‐analysis aimed to critically explore and quantify the evidence regarding the association between pain intensity and different body composition measures in adults suffering from CNLBP.

## 2 MATERIALS AND METHODS

### 2.1 Protocol and registration

This systematic review and meta‐analysis employed the Preferred Reporting Items for Systematic Reviews and Meta‐Analyses (PRISMA) checklist 2020^24^ and was registered on the International Platform of Registered Systematic Review and Meta‐analysis Protocols (INPLASY) (2022120064. doi: 10.37766/inplasy2022.12.0064).

### 2.2 Eligibility criteria

#### 2.2.1 Study designs

This study focused on study designs assessing associations between pain intensity and different body composition measures in adults suffering from CNLBP in primary studies such as cohort studies, case–control studies, cross‐sectional studies, as well as longitudinal or retrospective studies. Non‐peer‐reviewed manuscripts or conference abstracts, letters to authors, animal studies, and systematic reviews or meta‐analyses were excluded. Studies of all languages were included.

#### 2.2.2 Participants

The population of interest consisted of adults aged 18 years and older, suffering from CNLBP with no identified cause for LBP and persisting or fluctuating LBP with a minimum duration of 3 months.^10^, ^11^ LBP itself was defined as pain in the area of the lower margin of the twelfth ribs to the lower gluteal folds with or without pain in one or both legs.^25^ Specific causes, such as fractures, spinal stenosis, sciatica, spondylolisthesis, tumors, nerve root compression, disc hernias, and pregnancy‐, postpartum‐ or osteoporotic‐related LBP led to exclusion.^11^, ^26^ However, if the description of diagnosis for specific LBP was missing, it was considered as CNLBP, as previous studies showed difficulties defining CNLBP.^8^, ^16^ Studies including patients with combined upper and lower back pain were included, but patients with leg, neck, or thoracic pain alone were excluded.

All studies considered for this systematic review and meta‐analysis needed to report a sort of body composition measurement, for example, BMI, WHR, WC, hip circumference (HC), or any measure of fat mass or adipose tissue. BMI cut‐offs for the classification of normal weight, overweight, and obesity were used according to the World Health Organization (WHO) (normal BMI = 18.5–24.9 kg/m^2^, overweight = 25.0–29.9 kg/m^2^, and obesity ≥30 kg/m^2^).^27^ WC has been defined as a measure around the waist at the level of the midpoint between the lower margin of the last palpable rib and the top of the iliac crest, whereas the HC is measured around the widest portion of the buttock.^28^ However, other methods of measuring WC (e.g., measuring at the level of the umbilicus or at the smallest level of the torso) were included in the present study. Any fat mass or adipose tissue measurement investigated with DXA, BIA, ultrasound, or skinfold calipers was considered.^29^

Further, pain intensity had to be quantitatively assessed using a self‐reported Visual Analog Scale (VAS), Numeric (Pain) Rating Scale (NRS), or other assessments such as questionnaires that included pain intensity or severity.^30^

#### 2.2.3 Outcome

The primary outcome of this study includes the association between pain intensity and different body composition measurements in adults suffering from CNLBP.

Studies were included if they investigated at least one measure of body composition and its association with pain intensity. If only body composition and pain intensity were reported without exploring the association, authors were contacted for further calculations or raw data. When it was not possible to obtain this additional material, the study was excluded.

### 2.3 Information sources and search strategy

A systematic literature search was conducted on PubMed (National Library of Medicine, Bethesda, Maryland), CINAHL (via EBESCOhost), Embase (Elsevier), Web of Science (via Clarivate), and the Cochrane Library from database inception to January 2024. Medical Subject Headings (MeSH) and word variants were used for “back pain” and combined with terms for body composition like “overweight,” “obesity,” “body mass index,” or “waist‐hip ratio.” Table S1 shows the detailed search strategy for each database. Google Scholar was used to detect additional literature, using similar word variants as described above. Studies from different settings (participant characteristics, workplace settings, geographical regions) were considered for inclusion if they met the above‐mentioned inclusion criteria.

### 2.4 Study selection

All identified records from databases were organized using the Rayyan Web app.^31^ After removing duplicates, two reviewers (ML and MM) independently screened all titles and abstracts. After a first consensus meeting, full texts of relevant reports were collected and scrutinized for inclusion criteria by the same two reviewers independently. Exclusion reasons were reported, and in case of disagreements in these two steps, a consensus meeting with a third reviewer (AS) was held to discuss the final inclusion. The level of agreement was calculated using the ratio of similar‐rated studies to the total number of reviewed studies, and the results were expressed as percentages.

### 2.5 Study risk of bias assessment

To assess the methodological quality of the studies, the Critical Appraisal Skills Programme (CASP) checklists for cohort studies and case–control studies were defined in advance and listed in the INPLASY protocol.^32^ Items were rated with either “yes,” “no,” “can't tell,” or by a written justification. If an item did not apply to the study, it was rated as “not applicable.” Reasons for the rating in the comment section below the corresponding item offered the opportunity to critically analyze the study's content and to evaluate study validity, selection bias, measurement bias, classification bias, and confounding factors, as well as a description of results and the transferability of findings. Italicized hints were given for each question to corroborate the importance of the question. Two reviewers (ML and MM) rated the methodological quality independently. In cases of disagreement, they were resolved through discussion and, if necessary, by a third reviewer (AS).

### 2.6 Data collection and synthesis

Data were extracted and tabulated independently by two reviewers (ML and MM) and included information about (i) author, country, and publication year; (ii) study design; (iii) population characteristics (number of participants, sex, age); (iv) definition of CNLBP; (v) measures of body composition and pain intensity; and (vi) statistics (i.e., Pearson's correlation coefficient *r*, OR, Chi^2^‐statistic, regression coefficients *b*) and co‐variables/confounding factors (e.g., sex, age, education). If any clarification of methods or results was needed during data extraction, for example, in case of an unclear description of LBP duration, corresponding authors were contacted for clarification.

Meta‐analysis was performed when at least three studies evaluated the association between pain intensity and the same body composition measure (i.e., BMI, WHR, and WC). The effect size under evaluation in this present meta‐analysis was the association between pain intensity and the respective measure of body composition expressed as a Pearson correlation coefficient (*r*). This deviates from the protocol (2022120064), which initially anticipated using ORs or risk rations as effect size. However, during data extraction, it was determined that the correlation coefficient (*r*) was a more appropriate measure. In addition, adequate subgroups and sensitivity analyses were identified post hoc. For pain intensity, continuous values were used whenever possible (range of 0–10 or 0–100), while categorical measures of pain intensity were only accepted if no continuous values were available. In the case of missing correlation coefficients, other strategies were used to determine the effect size. For this purpose, the Chi^2^‐statistic, Kendall's tau‐b, and OR were converted into Pearson correlation coefficients according to Rosenberg^33^, Gilpin,^34^ and Borenstein,^35^ respectively. If associations were described with estimated marginal means, Cohen's *d* was calculated, following a conversion to Pearson's *r*.^35^ In addition, for studies that did not explicitly investigate the association between pain intensity and body composition measures but where authors provided raw data, the Pearson correlation coefficient *r* was calculated. Pooled weighted Pearson correlation coefficients were interpreted following the thresholds <0.20 = very weak, 0.20–0.39 = weak, 0.40–0.59 = moderate, 0.60–0.79 = strong, and >0.80 = very strong.^36^, ^37^, ^38^, ^39^, ^40^

The methodology as described in the Cochrane Handbook for Systematic Reviews of Interventions was followed in this study.^41^ For meta‐analyses, a random‐effects model with an inverse‐variance approach of DerSimonian and Laird was chosen, relying on Fisher's Z transformation of correlations.^41^ The hypothesis of no heterogeneity was tested using the *Q*‐statistic with its corresponding degrees of freedom and *p*‐value. In the case of statistically significant heterogeneity, potential sources (i.e., variability in participant characteristics, methods, and outcome measurement tools) were examined and further tested in subgroup analyses. The degree of heterogeneity, defined as the proportion of the total observed variation that can be explained by the between‐study variation, was expressed by Higgins' *I* ^2^ statistic and its 95% confidence interval (CI).^41^ Higgins' thresholds for the interpretation of *I* ^2^ were used (i.e., *I* ^2^ ≈25% = low, *I* ^2^ ≈50% = moderate, and *I* ^2^ ≥75% = high heterogeneity).^42^ Further, the likelihood of publication bias was evaluated by a visual (informal) inspection of asymmetry in the funnel plot and a calculation of Egger's regression coefficient with the intercept representing a measure of asymmetry.^43^, ^44^ To assess the consistency of results, sensitivity analyses were conducted by removing one study at a time. This approach allowed us to observe how each study individually influenced the overall findings and therefore to evaluate the robustness of the main findings in relation to the individual characteristics of the studies. A forest plot and corresponding CIs were established to visualize the pooling. Where no meta‐analysis was performed, the results were narratively summarized. All calculations were performed using R software, version 4.2.1, using the packages “effectsize” and “meta” (R Core Team, 2022).^45^

## 3 RESULTS

### 3.1 Study selection

**FIGURE 1:**
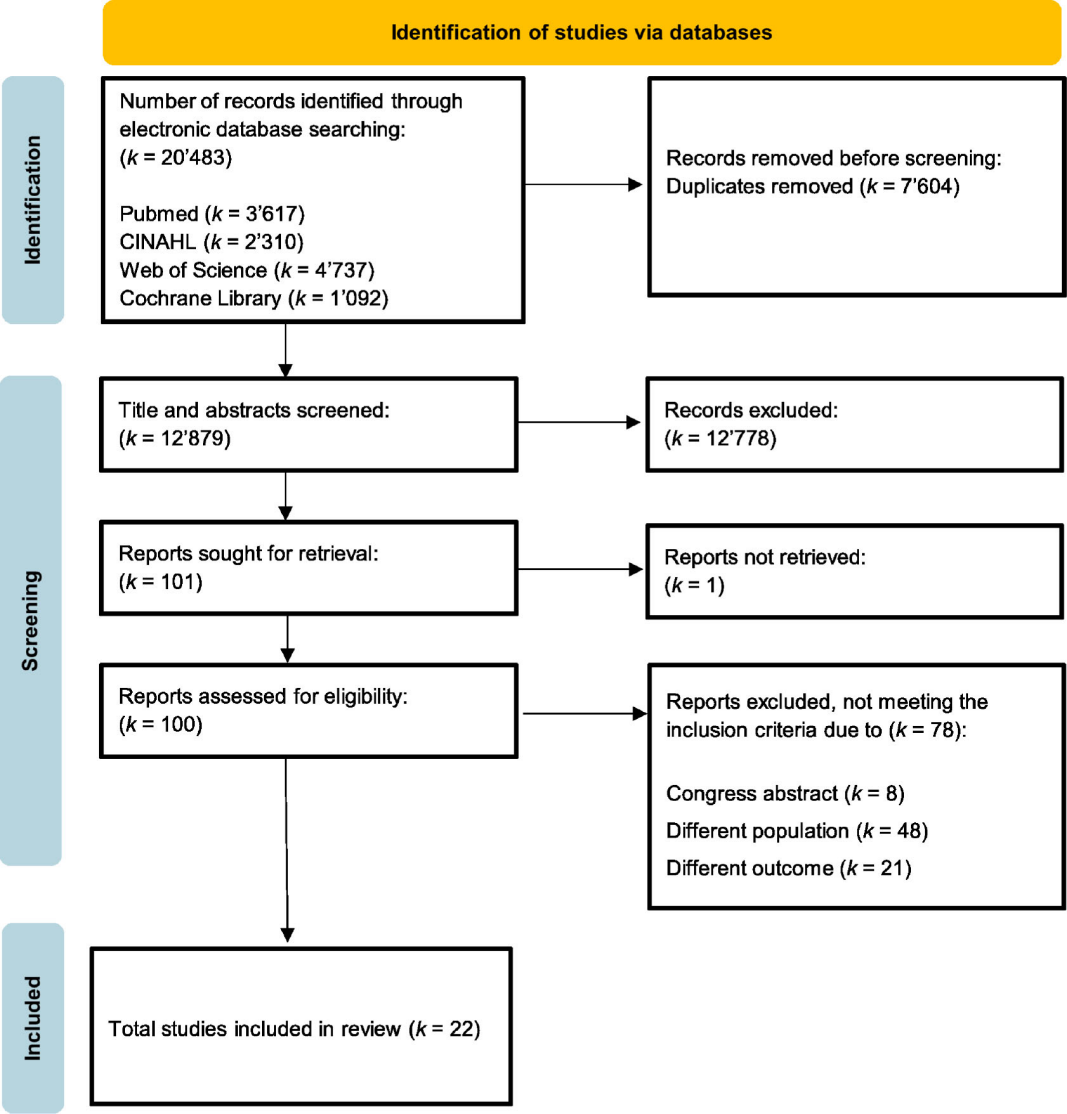
Preferred Reporting Items for Systematic Reviews and Meta‐Analyses (PRISMA) flowchart for study screening and identification of relevant studies.

A total of 20,483 records were retrieved from the literature search of all included databases until the 4th of January 2024 (Figure 1). After the removal of duplicates, 12,879 records were left for screening of titles and abstracts. Finally, 101 reports were evaluated, and of those, 79 were excluded because they did not match the inclusion criteria (*k* = 71) or were congress abstracts (*k* = 8). In total, 40 corresponding authors were contacted to ask for additional data that could not be extracted from the reports themselves or to provide more information about the study sample (i.e., if the sample had CNLBP). Nineteen authors responded, and eight of them provided the requested data and one provided an additional analysis. Detailed information on the authors contacted can be found in the Supporting Information. Due to insufficient minimal statistical requirements for calculating the effect size, the study by Radhika Rao^46^ was excluded at a late stage. In total, 22 reports were included, with participant sample sizes across studies ranging from 21 to 2101. For both screening stages, the level of agreement of the two reviewers (ML and MM) was 99.4% and 91.7%, respectively.

### 3.2 Study characteristics

Fifteen out of the 22 included reports used a cross‐sectional design^47^, ^48^, ^49^, ^50^, ^51^, ^52^, ^53^, ^54^, ^55^, ^56^, ^57^, ^58^ (four studies did not report the study design^58^, ^59^, ^60^, ^61^), while three were retrospective studies,^62^, ^63^, ^64^ three were prospective observational study designs,^65^, ^66^, ^67^ and one was a case–control study^68^ (Table 1). The studies were published between 2010 and 2023 and were conducted in Australia,^47^, ^48^, ^49^, ^65^ Benin,^54^ Brazil,^58^, ^66^, ^67^ Egypt,^59^ Greece,^57^ India,^53^ Italy,^63^, ^68^ Japan,^51^ Malaysia,^60^ Netherlands,^56^ Nigeria,^52^ Pakistan,^64^ Romania,^61^ Slovenia,^55^ Thailand,^50^ and the United States.^62^ Out of a total of 23,963 participants, the present meta‐analysis is based on 18,012 participants. Reasons for this discrepancy in sample size of the included studies and the meta‐analysis were (i) analysis on groups of only moderate or high pain intensity by the authors, (ii) because a minimal pain intensity threshold (e.g., NRS ≥ 1) was needed for pooling in the meta‐analysis, or (iii) because the study was not suitable for meta‐analysis due to missing information needed to calculate or convert the effect size. Seven studies focused on a specific CNLBP sample containing office workers,^60^ military police officers,^58^ female farmer workers,^67^ military service members,^56^ community‐dwelling older adults,^47^ low‐income sedentary females,^66^ and university‐level athletes.^53^

**TABLE 1:** Characteristics of included studies.

Study	Participants^a^ % female Mean age (SD)	Definition of CNLBP	Pain intensity	Body composition	Statistics, effect size
Ansari et al.^53^ Cross‐sectional study India	48 4.1% 22.13 years (2.82)	Pain >12 weeks, pain posterior from the lower margin of the twelfth ribs to the lower gluteal fold with or without referred pain in lower limbs	NRS (0–10) Current pain Pain last week	BMI (self‐reported)	Pain intensity current and BMI: Converted *r* ^c^ ^,^ ^d^ = 0.07 (CI: −0.34 to 0.46) Pain intensity last week and BMI: Converted *r* ^c^ ^,^ ^d^ = −0.03 (CI: −0.43 to 0.38) Adjustments for age, sex
Barros dos Santos et al.^66^ Descriptive correlational study Brazil	42 100% 50.33 years (11.67)	LBP duration ≥3 months, LBP pain intensity ≥4	VAS (0–10)	BMI (measured) WC	Pearson's *r* Pain intensity and BMI: *r* = 0.13 (CI: −0.18 to 0.42) *p* = 0.415
Brooks et al.^65^ Explorative study Australia	70 57.1% 39.57 years (11.01)	Pain between costal margin and gluteal fold, minimum duration of >3 months	VAS (0–100)	BMI (measured) WC HC WHR TBA Regional adiposity Adipositas ratios	Pearson's *r* Pain intensity and ratio between abdominal and lumbar adiposity thickness relative to BMI: *r* = 0.30 (CI: 0.07–0.50) *p* = 0.011
Carta et al.^68^ Case–control study Italy	115 81.7% 62.36 years (13.40)	CLBP, ≥3 months, exclusion of cancer, trauma, surgical intervention, degenerative neurological disease, acute tissue injury	NRS (0–10)	BMI (self‐reported)	Pain intensity and BMI: Converted *r* ^c^ ^,^ ^d^ = −0.06 (CI: −0.24 to 0.13) Adjustments for age, sex
Chou et al.^48^ Cross‐sectional study Australia	124 0% 62.9 years (14.0)	CPGQ, pain in past 6 months	CPGQ pain intensity score (0–100)	BMI (measured) WHR Fat mass (kg) Fat free mass (kg) Fat mass index (kg/m^2^) Fat free mass index (kg/m^2^) Fat mass/fat‐free mass ratio	Binary logistic regression, estimated marginal means Pain intensity and BMI: NA Converted *r* ^b^ = 0.10 (CI: 0.03–0.16) Adjustments for age, emotional disorder, education, mobility
Ferrari et al.^63^ Multicenter retrospective analysis Italy	310 61.3% 49.83 years (14.35)	Non‐specific LBP with or without referred pain, 12 months of median duration of LBP	NRS (0–10)	BMI (self‐reported)	Univariate linear regression analysis Pain intensity and BMI: *r* = 0.02 (CI: −0.09 to 0.13) *p* = 0.48
Gilmartin‐Thomas et al.^47^ Cross‐sectional study Australia	2101 66.2% 75.4 years (4.4)	Self‐developed pain questionnaire, moderate or severe LBP on most days LBP includes LBP alone and LBP in combination with upper back pain	NRS (0–10) Pain severity categories: mild, moderate, severe	BMI (measured)	Binary logistic regression OR of moderate or severe pain, healthy versus overweight F/M: 1.5 (CI: 1.27–1.76)/ 1.04 (CI: 0.84–1.30) OR of moderate or severe pain, healthy versus obese F/M: 2.91 (CI: 2.48–3.42)/ 2.23 (CI: 1.77–2.80) Moderate or severe pain, healthy versus overweight: converted *r* ^b^ = 0.05 (CI: 0.03–0.07) Moderate or severe pain, healthy versus obese: converted *r* ^b^ = 0.25 (CI: 0.23–0.28) Adjustments for age, depression
Hussien et al.^59^ Cross‐sectional study^f^ Egypt^f^	132 38% 33.11 years (9.23)	CNLBP, localized LBP, continuous or recurrent, duration >3 months	VAS (0–10)	BMI (measured) WC HC WHR	Kendall's *tau*‐b coefficient Pain intensity and BMI: τΒ = 0.05 *p* = 0.37 Converted *r* ^b^ = 0.08 (CI: −0.09 to 0.25)
Iizuka et al.^51^ Cross‐sectional study Japan	62 NA NA	Chronic LBP, duration >3 months, present pain	VAS (0–100)	BMI (measured) Muscle mass (kg) TBFM (kg) TBW (kg) Total body fat ratio (%) TBW ratio (%) Appendicular muscle mass (kg) Appendicular fat mass (kg) Trunk muscle mass (kg) Trunk fat mass (kg) Muscle mass index (kg/m^2^)	Simple and multiple linear regression models Pain intensity and BMI simple linear regression: *r* = −0.03 (CI: −0.12 to 0.07) Pain intensity and BMI multiple linear regression: *r* = −0.04 (CI: −0.13 to 0.06) Converted *r* ^b^ ^,^ ^d^ = −0.00 (CI: −0.25 to 0.25) Adjustments for age, sex in multiple linear regression analysis
Kastelic et al.^55^ Cross‐sectional study Slovenia	282 78.7% 52.31 years (12.96)	Occurrence of LBP in the last year (more than 90 days but not every day and daily)	VAS (0–100)	BMI (self‐reported)	Pain intensity and BMI: Converted *r* ^c^ ^,^ ^d^ = 0.24 (CI: 0.13–0.35) Adjustments for age, sex
Kossi et al.^54^ Observational cross‐sectional survey Benin	691 NA NA	Pain (NPRS > 0) between 12th rib and gluteal cleft, with or without radiation to the legs, ≥12 weeks, no specific underlying pathology or occurring episodically within 6 months	NRS (0–10)	BMI (measured)	Pain intensity and BMI: Converted *r* ^c^ = 0.06 (CI: −0.01 to 0.13)
Ojoawo et al.^52^ Cross‐sectional study Nigeria	64 100% 52.33 years (10.24)	Duration of LBP between 1 week up to 1 year. Non‐traumatic, non‐cancerous, non‐infectious, uncomplicated LBP with no radiation to lower limb, pain in lumbosacral region Authors confirmed that the participants had CNLBP >3 months duration	SDS (0–10)	BMI (measured) Weight Height WC HC % Body fat WHR Body density	Pearson's *r* Pain intensity and BMI: *r* = 0.54 (CI: 0.35–0.70) *p* <0.01
Prent et al.^56^ Cross‐sectional study Netherlands	21 14.3% 34.0 years (9.5)	LBP ≥12 weeks, average NRS in last 7 days ≥ 3, exclusion of specific spinal pathologies (e.g., radiculopathy, stenosis, spinal tumors)	NRS (0–10)	BMI (NA)	Pain intensity and BMI: Converted *r* ^c^ = 0.26 (CI: −0.19 to 0.62)
Sacomori et al.^67^ Descriptive study Brazil	30 100% NA	LBP and work questionnaire, constant pain >2 years duration and VAS ≥ 5	VAS (0–10)	BMI (measured)	Pain intensity and BMI: Converted *r* ^c^ = 0.25 (CI: −0.12 to 0.56)
Sakulsriprasert et al.^50^ Cross‐sectional study Thailand	30 70% 43.5 years (8.53)	CNLBP, unknown specific cause or pathology, ≥3 months duration, mild to moderate pain intensity (VAS 1–6)	VAS (0–10)	BMI (NA)	Spearman's *Rho* ^i^ Pain intensity and BMI: *rs* = −0.05 (CI: −0.40 to 0.32) *p* = 0.794
Shariat et al.^60^ Cross‐sectional study^f^ Malaysia	752 NA NA	CMDQ, chronic pain^g^	CMDQ Pain severity categories: low, medium, high	BMI (self‐reported) BMI (kg/m^2^) categories: ≤18.4 18.5–24.99 25–29.99 ≥30	Chi^2^ test Pain severity and BMI: Chi^2^ = 5.79 *p* = 0.047 Converted *r* ^b^ = 0.09 (CI: 0.02 to 0.16)
Siddiqui et al.^64^ Retrospective observational study Pakistan	31 NA NA	Chronic LBP >3 months, inclusion of different diagnoses containing non‐specific back pain	NRS (0–10)	BMI (self‐reported)	Pain intensity and BMI: *R* ^e^ = 0.32 (CI: −0.04 to 0.61) *p* = 0.081
Sirbu et al.^61^ Cross‐sectional study^f^ Romania	76 73.7% 53.79 years (13.82)	CLBP with pain in back, between last rib and gluteal fold, mechanical characteristics, >3 months duration, exclusion of specific causes for LBP	VAS (0–10)	BMI (measured)	Spearman's *Rho* ^i^ Pain intensity and BMI: *rs* = −0.15 *p* > 0.05
Tavares et al.^58^ Cross‐sectional study^f^ Brazil	79 0% NA	CNLBP, >3 months duration	VAS (0–10)	BMI (measured)	Multiple regression analysis^h^ Pain intensity and BMI: Moderate pain intensity group: *b* = 0.049 (CI: −0.07 to 0.168) *p* = 0.41 Severe pain intensity group: *b* = 0.001 (CI: −0.072 to 0.071) *p* = 0.99
Tsatsaraki et al.^57^ Cross‐sectional study Greece	253 62% 59.30 years (15.0)	CLBP, exclusion of lumbar spine fracture or surgery	VAS (0–10)	BMI (measured)	Pain intensity and BMI: Converted *r* ^c^ ^,^ ^d^ = −0.03 (CI: −0.15 to 0.10) Adjustments for age
Urquhart et al.^49^ Cross‐sectional study Australia	135 83.1% 47.4 years (9.0)	CPGQ, pain intensity over the past 6 months	CPGQ (0–100)	BMI (measured) Android fat mass Gynoid fat mass Lean tissue mass total	Ordinal logistic regression and multivariate analysis^h^ Pain intensity and BMI univariate analysis: OR = 1.38 (CI: 1.12 to 1.68) *p* = 0.002 Pain intensity and BMI multivariate analysis: OR = 1.35 (CI: 1.09 to 1.67) *p* = 0.005 Adjustments for age, sex, height, physical activity in multivariate analysis
Wood et al.^62^ Retrospective study United States	175 59.1% 50.95 years (15.05)	Lower back pain, >3 months duration	NRS (0–10) Pain intensity categories: 0–3 = low 4–6 = medium 7–10 = high	BMI (measured)	Pearson's *r* Pain intensity and BMI: *r* = 0.13 (CI: −0.02 to 0.27) *p* = 0.098

*Note*: Data was extracted concerning the research question and may not contain all investigated variables of the individual studies.

Abbreviations: BMI, body mass index (kg/m^2^); CI, 95% confidence interval; CLBP, chronic low back pain; CMDQ, Cornell Musculoskeletal Discomfort Questionnaire; CNLBP, chronic non‐specific low back pain; CPGQ, Chronic Pain Grade Questionnaire; F, females; HC, hip circumference; LBP, low back pain; M, males; NA, not applicable; NRS, Numerical Rating Scale; OR, odds ratio; SDS, Semantic Differential Scale; TBA, total body adiposity percentage; TBFM, total body fat mass; TBW, total body water; VAS, Visual Analog Scale; WC, waist circumference; WHR, waist–hip ratio; y, year; *b*, slope in multiple regression.

^a^ Only data of the population of interest, that is, available data of CNLBP.

^b^ Conversion to Pearson correlation coefficient, as described in Section 2.

^c^ Calculation of Pearson correlation coefficient based on raw data.

^d^ Raw and adjusted values were not of big difference; therefore, only raw values were used in this analysis for comparison with other study results.

^e^ Authors provided a calculation of the association between the NRS Score and BMI in the CNLBP subgroup.

^f^ This was not mentioned in the study.

^g^ Authors were contacted for clarification of LBP chronicity, but no definition was given.

^h^ No conversion to Pearson correlation is possible due to missing additional information from authors, therefore not included in the meta‐analysis.

^i^ Spearmen correlation coefficient was handled as a Pearson correlation coefficient in meta‐analysis.

In 18 out of the 22 studies, NRS or VAS was used to assess pain intensity.^47^, ^50^, ^51^, ^53^, ^54^, ^55^, ^56^, ^57^, ^58^, ^59^, ^61^, ^62^, ^63^, ^64^, ^65^, ^66^, ^67^, ^68^ Two studies used the Chronic Pain Grade Questionnaire (CPGQ),^48^, ^49^ and one study used the Semantic Differential Scale (SDS), each resulting in a score between 0 and 10.^52^ Only one study used the Cornell Musculoskeletal Discomfort Questionnaire (CMDQ) with categorial classification for pain intensity.^60^ All included studies investigated BMI as a measure of body composition. BMI was calculated based on weight and height measurements conducted by assessors in 14 studies.^47^, ^48^, ^49^, ^51^, ^52^, ^54^, ^57^, ^58^, ^59^, ^61^, ^62^, ^65^, ^66^, ^67^ In six studies, BMI was self‐reported by the participants,^53^, ^55^, ^60^, ^63^, ^64^, ^68^ and in two studies, no description was found for the collection of BMI.^50^, ^56^ Four studies reported WHR measures^48^, ^52^, ^59^, ^65^ and WC measures^52^, ^59^, ^65^, ^66^, respectively. DXA measurements were reported in two studies,^48^, ^49^ while ultrasound,^65^ BIA,^51^ or skinfold caliper measures^52^ were reported in three separate studies, respectively.

### 3.3 Quality appraisal

**FIGURE 2:**
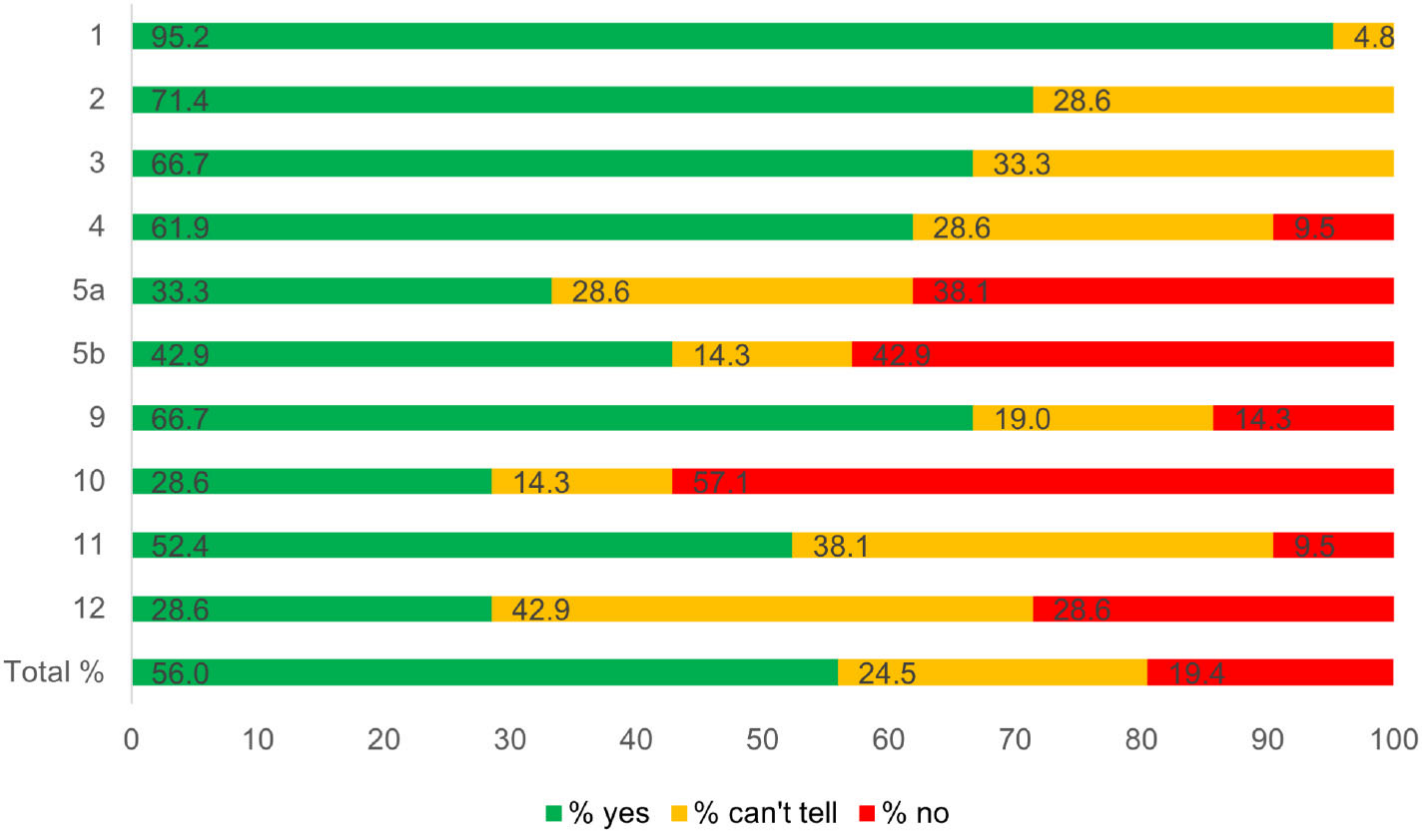
Quality appraisal of Critical Appraisal Skills Programme (CASP) checklist for cohort studies. *Legend:* Items 7 and 8 are addressed in Chapter 3.3, as they do not have a rating section, only a comment section. Items 6a and 6b are not listed, as none of the included studies performed a follow‐up. One study was rated with the CASP checklist for case–control studies and is summarized in Chapter 3.3.

The CASP checklist for cohort studies was used in 21 studies, revealing that 56% of the items were rated with “yes.” Figure 2 shows the different percentages reached for each item, and Table S2 depicts a detailed overview of the quality appraisal rating of all included studies. Ten of 21 studies accurately described the recruitment strategy and the measurement of exposure and outcome (Items 1–4), receiving ratings with “yes” only. Substantial limitations were found for confounding factors (e.g., age, sex, education), which were not considered in the design or analysis in 12 of 21 studies (Items 5a and 5b). The reason for this critical rating can be attributed to the study designs, which were mainly of cross‐sectional or retrospective nature. The credibility of results (Items 7–9) was lacking in seven studies due to limited methodological description, no CIs depicted, small sample sizes, no distinguishing chronic from non‐chronic LBP, and lack of transparent presentation of results (e.g., in tables). Overall, no clear conclusion has been drawn regarding the transferability of study findings, as the study designs included (i.e., cross‐sectional designs and observational studies) were not appropriate for such a statement (Items 10–12).^69^ Further, no single study performed a follow‐up analysis (Items 6a and 6b). The single study that was rated with the CASP checklist for case–control studies showed limitations in adjusting for confounding factors, transferability of study findings, and poor presentation of statistical results.

### 3.4 Association between pain intensity and body composition

**FIGURE 3:**
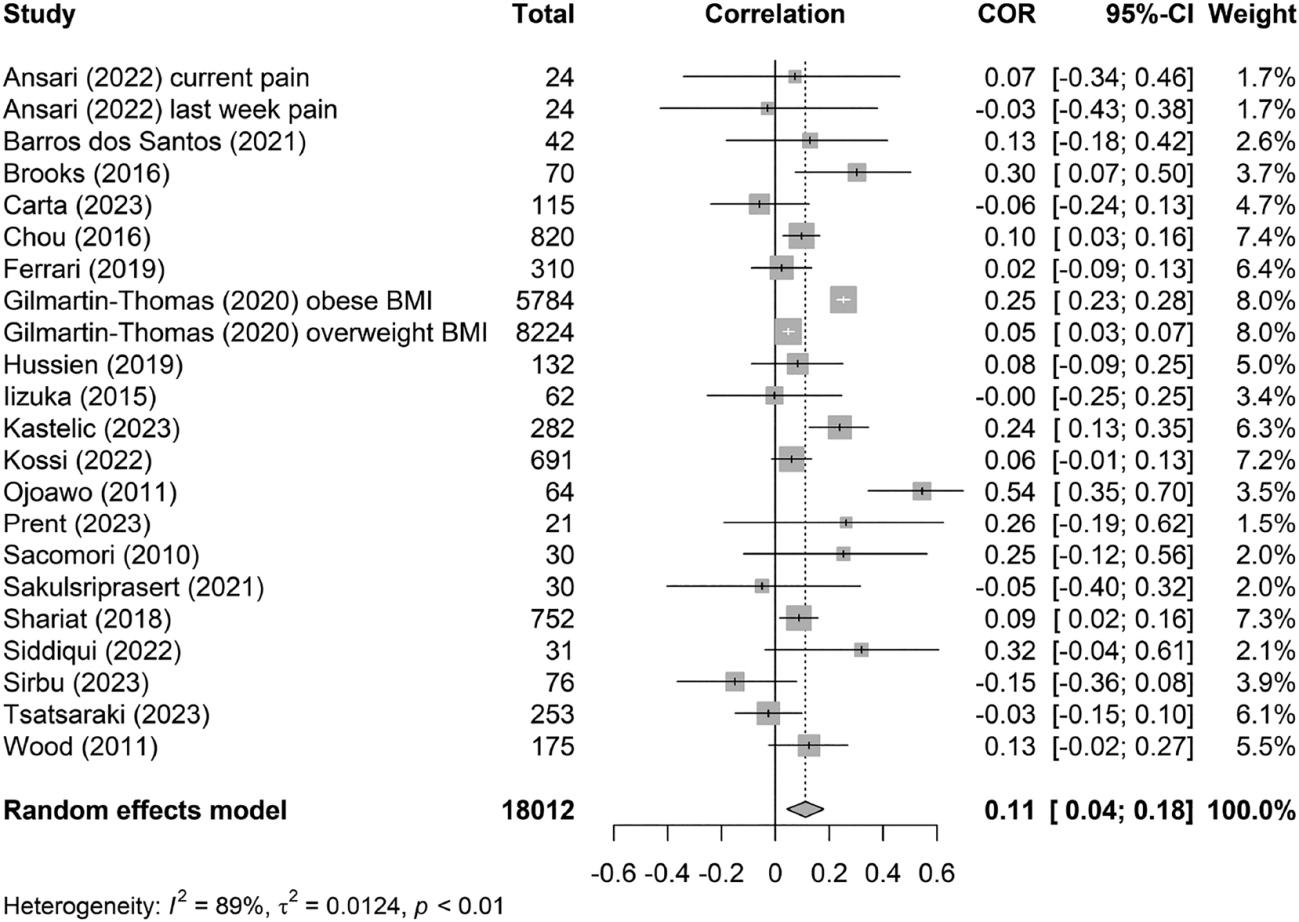
Forest plot of the pooled correlation of pain intensity and body mass index (BMI).

Meta‐analysis of the association between pain intensity and BMI in people suffering from CNLBP found a pooled weighted positive, very weak Pearson correlation coefficient of 0.11 (95% CI: 0.04–0.18) (Figure 3). A total of 20 studies were included in the analysis, compromising 18,012 participants. This finding indicates that higher levels of pain intensity were very weakly associated with a higher BMI in people with CNLBP. Between studies, heterogeneity was statistically significant (*Q*‐statistic = 199.96, *df* = 21, *p* < 0.0001) and considerable (*I* ^2^ = 89.5%, 95% CI: 85.5–92.4). Visual inspection of the funnel plot (Figure S1) and the intercept value from Egger's test (*t* = −0.21, *p* = 0.83) suggested no presence of asymmetry of the data in the meta‐analysis, that is, no evidence of publication bias.

**FIGURE 4:**
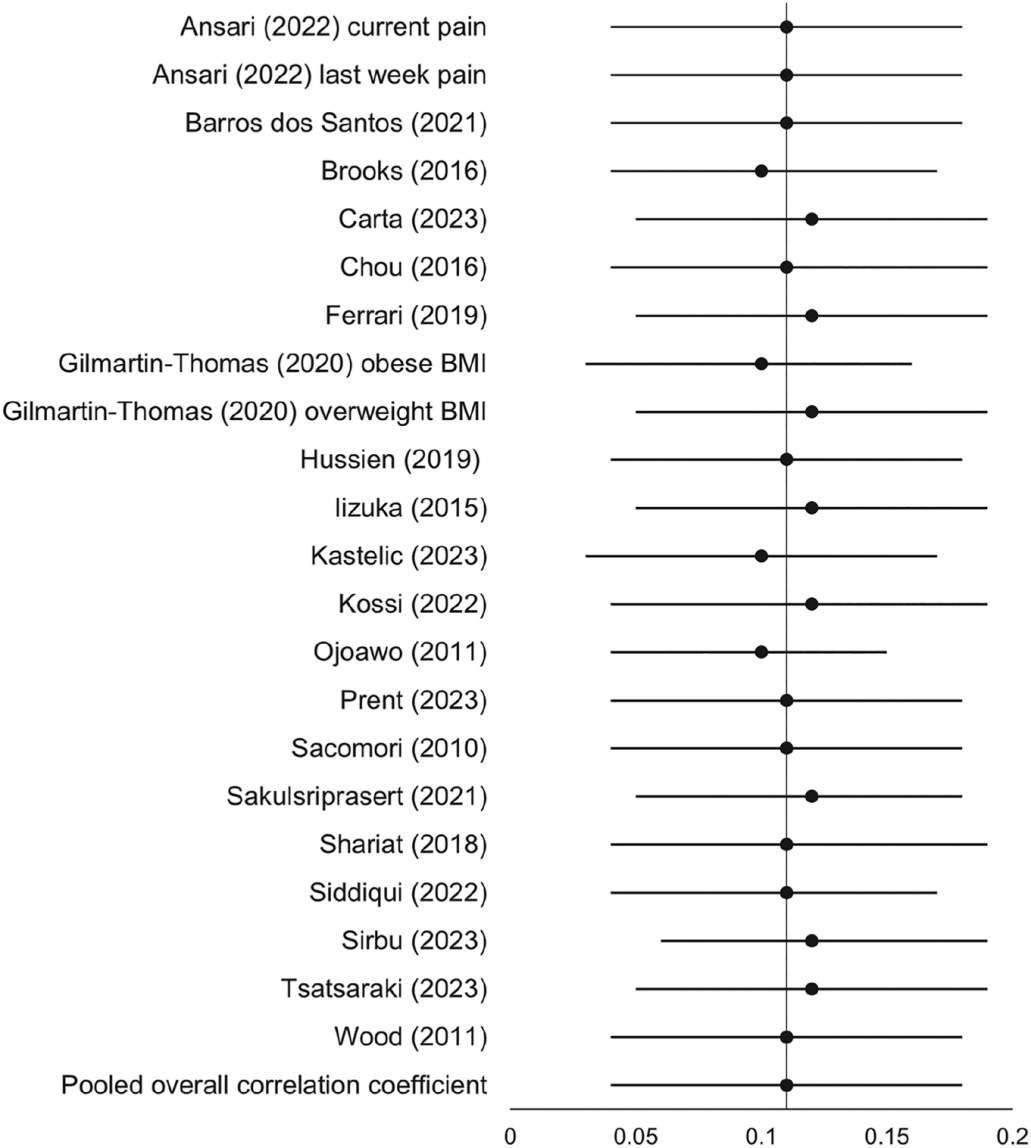
Sensitivity analysis: Meta‐analysis estimates, with named study omitted from analysis. *Legend:* Dots: effect size; Whiskers: 95% confidence intervals with upper and lower limits; Pooled overall correlation coefficient: indicates the pooled correlation coefficient for the meta‐analysis including all studies investigating pain intensity and BMI; Vertical line: represents the value of the overall meta‐analysis (*r* = 0.11).

A sensitivity analysis was conducted to assess the robustness of the overall weighted mean correlation coefficient against individual studies (e.g., small studies or negative effect sizes) by removing every study stepwise from the meta‐analysis. The pooled correlation coefficient did not alter remarkably, with small absolute differences between pooled correlation coefficients of the overall meta‐analysis and the sensitivity analyses (differences in pooled *r*‐values of ±0.01) (Figure 4). Because sensitivity analyses did not seem to considerably impact the overall result, revealing very weak positive correlation coefficients between pain intensity and BMI in people with CNLBP, all studies were included in the final analysis.

**FIGURE 5:**
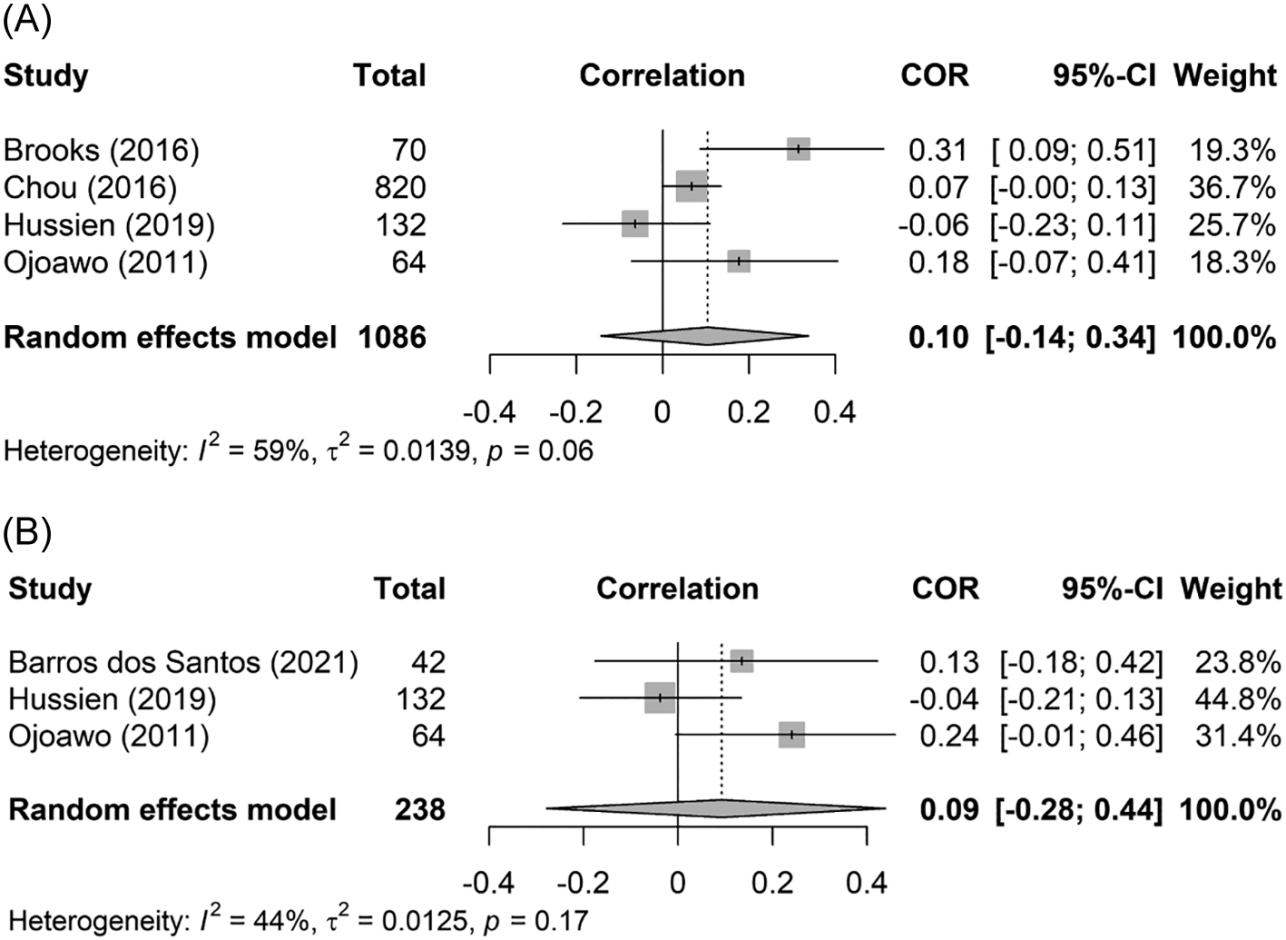
Forest plot of the correlation between pain intensity and waist–hip ratio (WHR) (A) and pain intensity and waist circumference (WC) (B).

A meta‐analysis of studies investigating correlations between pain intensity and WHR (*k* = 4) totaling 1086 participants showed a positive, very weak pooled weighted Pearson correlation coefficient of 0.10 (95% CI: −0.14 to 0.34) (Figure 5A). Heterogeneity was statistically not significant and moderate (*Q*‐statistic = 7.39, *df* = 3, *p* = 0.06; *I* ^2^ = 59%, 95% CI: 0.0 to 86.5).

The pooled weighted correlation coefficient between pain intensity and WC (*k* = 3), totaling 238 participants, was positive but very weak (*r* = 0.09, 95% CI: −0.28 to 0.44) (Figure 5B). Heterogeneity was statistically not significant and low (*Q*‐statistic = 3.56, *df* = 2, *p* = 0.17; *I* ^2^ = 44%, 95% CI: 0.0 to 83.2). No pooled association between pain intensity and any other body composition characteristics could be calculated because the number of studies was too small.

### 3.5 Pooled correlations in subgroup analysis by characteristics of studies

Subgroup analyses were performed for the overall meta‐analysis based on the primary outcome of the association between pain intensity and BMI, collection of BMI (measured vs. self‐reported), categories of BMI, pain intensity, sex, age, and definition of CNLBP (Table 2).

**TABLE 2:** Pooled correlation coefficient by subgroups with different characteristics.

	*N* participants	Pooled correlation (95% CI)	*I* ^2^ (%)
			(95% CI)
Categorization of BMI:			
BMI 18.5–24.9 kg/m^2^	1194	0.04 (−0.01–0.08)	0 (0.0–74.6)
BMI 25–29.9 kg/m^2^	10,899	0.14 (0.02–0.25)	74 (54.3–85.4)
BMI ≥30 kg/m^2^	5826	0.25 (0.12–0.37)	0 (NA)
BMI self‐reported	1538	0.09 (−0.02–0.21)	51 (0.0–79.4)
BMI measured	16,423	0.12 (0.02–0.23)	94 (90.6–95.5)
Pain intensity:			
Low (mean score < 4)	901	0.07 (−0.02–0.16)	0 (0.0–74.6)
Moderate to high (mean score ≥ 4)	16,227	0.13 (0.03–0.23)	93 (90.2–95.3)
Sex:			
Female	136	0.34 (−0.28–0.76)	67 (0.0–90.5)
Age:			
Mean age ≥ 50 years	15,835	0.12 (−0.02–0.26)	95 (92.8–96.7)
Mean age < 50 years	1363	0.08 (0.02–0.14)	0 (0.0–67.6)
Association pain intensity and BMI as:			
Primary outcome	16,572	0.13 (0.03–0.22)	93 (89.7–95.1)
Not primary outcome	1440	0.08 (−0.03–0.19)	52 (0.0–78.3)
Definition of CNLBP:			
CNLBP (no specific cause)	1647	0.11 (−0.05–0.27)	69 (37.5–84.4)
CNLBP (pain duration)	16,289	0.14 (0.08–0.20)	93 (89.9–95.4)

Abbreviations: BMI, body mass index; CI, confidence interval; CNLBP, chronic non‐specific low back pain; *N* participants, number of participants; NA, not applicable.

A subgroup meta‐analysis of studies was conducted, comparing those that investigated the association between pain intensity and BMI as primary objective^47^, ^48^, ^50^, ^51^, ^52^, ^59^, ^60^, ^61^, ^62^, ^63^, ^64^, ^65^, ^66^ with those that did not investigate this association as primary objective.^53^, ^54^, ^55^, ^56^, ^57^, ^67^, ^68^ Both showed very weak, positive associations (*r* = 0.08, 95% CI: −0.03 to 0.19, *I* ^2^ = 52% vs. *r* = 0.13, 95% CI: 0.03 to 0.22, *I* ^2^ = 93%, respectively). Furthermore, studies were grouped according to measured BMI versus self‐reported BMI, both resulting in a positive, very weak association between pain intensity and BMI in persons with CNLBP when BMI was measured by assessors (*r* = 0.12, 95% CI: 0.02 to 0.23, *I* ^2^ = 94%)^47^, ^48^, ^51^, ^52^, ^54^, ^57^, ^59^, ^61^, ^62^, ^65^, ^66^, ^67^ compared with self‐reported BMI (*r* = 0.09, 95% CI: −0.02 to 0.21, *I* ^2^ = 51%).^53^, ^55^, ^60^, ^63^, ^64^, ^68^

In addition, the mean BMI of study participants was categorized according to the WHO guidelines to normal, overweight, and obese BMI (18.5–24.9 kg/m^2^, 25–29.9 kg/m^2^, ≥30 kg/m^2^, respectively).^27^ Meta‐analysis on the association between pain intensity and normal BMI in individuals with CNLBP showed a positive, very weak weighted correlation coefficient (*r* = 0.04, 95% CI: −0.01 to 0.08, *I* ^2^ = 0%),^50^, ^53^, ^54^, ^63^, ^68^ while a BMI classified as overweight^47^, ^48^, ^52^, ^55^, ^56^, ^57^, ^59^, ^60^, ^61^, ^62^, ^65^, ^67^ or obese^47^, ^66^ showed positive, very weak to weak pooled weighted correlation coefficient (*r* = 0.14, 95% CI: 0.02 to 0.25, *I* ^2^ = 74% and *r* = 0.25, 95% CI: 0.12 to 0.37, *I* ^2^ = 0%, respectively). The subgroup analysis investigating the association between low mean pain intensity (mean pain score < 4)^50^, ^51^, ^53^, ^54^, ^65^ and moderate to high mean pain intensity (mean pain score ≥ 4)^47^, ^48^, ^52^, ^55^, ^56^, ^57^, ^61^, ^62^, ^63^, ^64^, ^66^, ^67^, ^68^ and BMI, each showed positive, very weak associations (*r* = 0.07, 95% CI: −0.02 to 0.16, *I* ^2^ = 0% and *r* = 0.13, 95% CI: 0.03 to 0.23, *I* ^2^ = 93%, respectively).

Studies including female participants only (*n* = 136),^52^, ^66^, ^67^ showed a positive but weak association between pain intensity and BMI (*r* = 0.34, 95% CI: −0.28 to 0.76, *I* ^2^ = 67%). Due to low numbers of studies, no analysis was performed for male participants (*k* = 2).

Further, a subgroup analysis was performed for studies with a mean age of participants ≥50 years^47^, ^48^, ^52^, ^55^, ^57^, ^61^, ^62^, ^66^, ^68^ and <50 years.^50^, ^53^, ^56^, ^59^, ^60^, ^63^, ^65^ In both age‐categories, the pooled weighted correlation coefficients between pain intensity and BMI were positive and very weak (≥50 years: *r* = 0.12, 95% CI: −0.02 to 0.26, *I* ^2^ = 95%, and <50 years: *r* = 0.08, 95% CI: 0.02 to 0.14, *I* ^2^ = 0%, respectively).

A last subgroup analysis was conducted to compare studies defining CNLBP as “non‐specific” or explicitly excluding specific causes (e.g., fracture, radiculopathy)^50^, ^52^, ^54^, ^56^, ^57^, ^59^, ^61^, ^63^, ^64^, ^68^ with those defining it solely by pain duration.^47^, ^48^, ^51^, ^53^, ^55^, ^60^, ^62^, ^65^, ^66^, ^67^ Both analyses showed a positive, very weak association between pain intensity and BMI (CNLBP defined by no specific cause: *r* = 0.11, 95% CI: −0.05 to 0.27, *I* ^2^ = 69% and CNLBP defined by pain duration: *r* = 0.14, 95% CI: 0.08 to 0.20, *I* ^2^ = 93%).

### 3.6 Results of studies excluded from meta‐analysis

Given the inability to extract required data from the publication to convert OR to a Pearson's *r* (because of missing information on BMI values) and no answer was received from authors, the study by Urquhart et al. was not included in the final meta‐analysis.^49^ This study showed a clinically relevant and statistically significant association between pain intensity and BMI (OR = 1.35, 95% CI: 1.09 to 1.67, *p* = 0.005), indicating that individuals with high BMI values have 35% higher odds for experiencing higher pain intensity. The same authors found that in persons with CNLBP, greater fat mass but not lean tissue mass seemed to be positively associated with pain intensity when measured using DXA.^49^ In addition, the study of Tavares et al. was not included in the meta‐analysis as no information from the authors was received to convert regression coefficients to a Pearson's *r* from the publication's data.^58^ This study showed no statistically significant associations between BMI and moderate (*b* = 0.049, 95% CI: −0.07 to 0.168, *p* = 0.410) or severe pain intensity (*b* = 0.001, 95% CI: −0.072 to 0.071, *p* = 0.992).

For other body composition measures, which were not included in meta‐analysis, positive, weak, and statistically significant correlation coefficients were found between pain intensity and the total subcutaneous and visceral abdominal adiposity thickness relative to lumbar adiposity thickness (*r* = 0.32, *p* = 0.007, *n* = 70), which was measured by ultrasound.^65^ The same authors found that 9.1% of the variance in pain could be explained by this ratio.^65^ Another study used skinfold thickness measurements as a body composition outcome measure and reported a positive, high and statistically significant correlation coefficient between pain intensity rating and body fat percentage in 64 women suffering from CNLBP (*r* = 0.67, *p* < 0.01) and a positive, moderate, and statistically significant association (*r* = 0.41, *p* < 0.05) between pain intensity and HC.^52^ Another study using BIA as a body composition measure, concluded that none of the included body composition parameters (i.e., appendicular fat mass, trunk fat mass as well as total body fat ratio or lean tissue masses) was statistically significantly associated with pain intensity.^51^ This was also reported in one study using DXA as measure for body composition, which showed no statistically significant difference in fat mass, fat mass index, fat‐free mass, and fat‐free mass index in men suffering from CNLBP with high pain intensity, compared with the group of men with no or low pain intensity.^48^

## 4 DISCUSSION

### 4.1 General interpretation of results in context of other evidence

The present systematic review and meta‐analysis showed positive, very weak associations between pain intensity and BMI, WHR, and WC in adults with CNLBP. Therefore, the present synthesis showed that there is some but limited evidence suggesting that a higher level of pain intensity is very weakly associated with higher BMI, WHR, and WC values in adults suffering from CNLBP.

The categorization of BMI into normal, overweight, and obese revealed a marginally stronger association between pain intensity and increasing BMI in such patients with CNLBP (*r* = 0.04 for normal BMI, *r* = 0.14 for overweight BMI, and *r* = 0.25 for obese BMI), suggesting a minor dose–response relationship. This result corroborates the findings of other studies, including adult and adolescent participants, which found higher associations between CLBP and overweight or obese BMI compared with normal BMI.^8^, ^70^ A survey from the United States, which assessed the association between any physical pain that occurred yesterday and obesity in one million people, concluded that from each BMI categorization greater than normal (≥25 kg/m^2^), a higher probability of reporting pain was observed.^71^

Different studies reported a statistically significant association between overweight or obesity and an increased risk of CLBP in adults and adolescents.^7^, ^8^, ^16^ All mentioned studies, except You et al., used only BMI as a measure of overweight and obesity.^16^ Importantly, BMI alone does not account for body fat distribution,^16^, ^22^ but is widely acknowledged as a measure of adiposity, and literature for positive correlations between BMI and body fat percentage is evident.^72^, ^73^ You et al. pointed out, that persons with higher body fat rate, WHR, WC, or total body fat mass were at increased risk of LBP, regardless of BMI values.^16^ Thus, measurements focusing on distribution and amount of body fat might identify people with obesity better than BMI, which focuses on body weight.^16^ For a comprehensive evaluation, this systematic review included BMI as well as other measures for body composition, but no pooling for fat mass, body fat percentage, HCs, or subcutaneous adipose tissue thickness was possible, due to the small number of studies.

Overweight and obesity might be a leading risk factor for CLBP, but the very weak association between pain intensity and BMI, WHR, and WC found in this review and meta‐analysis suggests that overweight and obesity measures do not solely account for pain intensity levels in persons who suffer from CNLBP. Most included studies were cross‐sectional designed, limiting the ability to determine a temporal relationship between exposure and outcome. The observed association between pain intensity and body composition measures might be bidirectional (e.g., higher pain intensity may contribute to a higher BMI and vice versa), precluding any causal conclusions. Given the very weak association found, these results should be interpreted cautiously, as the methodology of the included studies limits not only the ability to infer causation but also to account for confounding factors that may affect the robustness of the observed association. While a very weak positive association is suggested, the methodological constraints of these studies reduce confidence in the strength and direction of the association.

Different theories for the explanation of mechanisms for pain related to overweight or obesity in adults have been proposed. First, the impact of excess weight results in mechanical pressure on skeletal muscles, the spine (spinal discs and cord), and other joints, which might contribute to LBP.^74^, ^75^ First, from a biomechanical perspective, people with obesity are at greater risk for lifting‐related LBP.^74^ The excess weight may impair the nutrition supply to intervertebral discs and surrounding tissue, due to the increased load on the structures.^75^ Second, adipose tissue functions as an endocrine organ^76^ by releasing pro‐inflammatory cytokines,^21^, ^77^, ^78^ which are significantly elevated in individuals with obesity compared with those with normal weight.^79^ Very weak to strong positive associations have been observed between pro‐inflammatory cytokine levels and BMI, visceral adiposity, WC, and adipose body mass (kg),^79^, ^80^ underlining the endocrine function of adipose tissue and its possible contribution to systemic low‐grade inflammation.^77^, ^81^

Third, those proinflammatory cytokines may also facilitate neuropathic pain and hyperalgesia, characterized by a higher sensitivity to mechanical or heat stimuli.^82^ McKendall and Haier showed that persons with overweight and obesity had higher pain sensitivity compared with persons with normal weight, corroborating the theory of inflammatory pain.^83^ Furthermore, inflammatory processes lead to peripheral and central pain sensitization, which may contribute to pain chronification in patients on the one hand and to persistent pain in people suffering from CNLBP on the other hand.^84^, ^85^, ^86^

Fourth, research showed that fear‐avoidance beliefs, pain‐related fear, and pain anticipation resulted in poor physical and behavioral performances due to avoidance of physical activity.^87^, ^88^, ^89^ Physically inactive persons with obesity were at the highest risk of radiating LBP compared with those who were physically active.^90^ In combination with an exceeding energy intake due to physical inactivity, this could lead to weight gain.^91^

The described mechanisms could probably explain why overweight and obesity are a risk factor or a maintaining factor for LBP that could lead to chronicity. However, this meta‐analysis only found very weak associations between pain intensity and overweight or obesity in addition to substantial risk of bias, implying other factors need to be apparent to explain pain intensity in this sample. Literature shows that pain intensity itself is considered a risk factor for CNLBP.^92^ In 2023, a cross‐sectional study described that central sensitization, measured by the Central Sensitization Inventory, is suggested as an underlying mechanism of pain in patients with CLBP.^93^ Further, other aspects such as anxiety, kinesiophobia, and depression seem to be associated with pain intensity in patients with CLBP and CNLBP.^94^, ^95^ In addition, disability, socioeconomic status, sex, and age seem to be associated with pain intensity in patients with chronic pain in general.^96^ Overall, the prevalence of LBP seems to increase with age with a peak around 85 years and is higher in females compared with males.^8^, ^10^, ^97^ According to the World Obesity Atlas, adult women reported higher numbers of obesity in 2020 than men.^98^ Further, women are more sensitive to noxious stimulation than men in general,^99^ which might have resulted in general higher ratings of pain intensity. Subgroup analyses of this study revealed a slightly higher correlation between pain intensity and BMI in adults being 50 years of age or older compared with adults of younger age (<50 years) (*r* = 0.12 and *r* = 0.08, respectively) and a higher correlation coefficient for the association between pain intensity and BMI including female‐only studies compared with the overall meta‐analysis of studies including all genders (*r* = 0.34 vs. *r* = 0.11). Therefore, the results of these meta‐analyses corroborate previous findings, but it should be noted that the results may be biased by the small sample sizes of the studies included in the gender‐specific sub‐group analysis.

Clinical guidelines recommend the assessment of prognostic factors like pain severity, psychological distress, and work‐related factors and recommend a multidisciplinary biopsychosocial rehabilitation for people suffering from CNLBP.^100^, ^101^ However, there is no recommendation to assess body composition or to integrate weight management in the treatment of CNLBP.^102^ Based on this systematic review with meta‐analysis, only a very weak, positive association was found between pain intensity and body composition, and no recommendations for future therapeutic management can be made. Despite this, a small proof‐of‐concept study showed promising results regarding the integration of a weight loss program to exercise therapy, resulting in reduced disability and 48% of pain reduction.^103^ Therefore, more experimental research is needed to explore potential associations between pain intensity and different body composition measurements.

### 4.2 Strengths and limitations

This present study has some strengths. First, this systematic review and meta‐analysis is, to the best of the authors' knowledge, the first meta‐analysis investigating pain intensity and its association with body composition measures in adults with overweight and obesity suffering from CNLBP. Second, a broad definition of CNLBP was used, as the term “low back pain” describes a symptom and was defined differently among studies. Therefore, studies that investigated patients suffering from CLBP but excluded specific causes for LBP were handled as CNLBP, leading to a sensitive systematic literature research. This allowed the inclusion of different samples, which denies a generalizability but opened a possibility to show rather consistent results among different samples. Further, multiple body composition measures were investigated, making the association between pain intensity and overweight and obesity more credible. Finally, sensitivity and subgroup analyses were performed to evaluate the robustness of the meta‐analyzed results against different moderating factors.

However, there are also some limitations to mention. Most of the reviewed studies were cross‐sectional, and no cohort study or randomized controlled trial was included in the final review. This led to a generally lower methodological quality rating, as the CASP tool for cohort and case–control studies was used. During the consensus meetings, it emerged that one rater (ML) consistently rated the studies more critically than the other rater (MM). In the vast majority of cases, the more critical grading was applied. Further, the quality rating must be interpreted with caution for the eight included reports for which additional material was provided by the authors. The population or the outcome of these studies did not fit the initial research question, which affected the quality rating of different CASP items.

Only nine of the 22 studies included in this systematic review addressed or identified confounding factors. From those studies, the adjusted correlation coefficients were comparable to the raw values. To enable a consistent comparison between studies, it was decided to use raw correlation coefficients as a measure of effect size.

Substantial heterogeneity was observed in the meta‐analyzed correlation coefficients (*I* ^2^ = 89%). Subgroup analyses failed to explain this heterogeneity, likely due to the low number of studies per subgroup (see Table 2). However, the variability in sample characteristics, such as age, gender, occupation, and the distribution of BMI and pain intensity (see Table 1), may partly explain the heterogeneous effect sizes. Furthermore, differences in the methods used to assess BMI and pain intensity across studies could have resulted in either under‐ or overestimation of these variables. These limitations may reduce the external validity of the findings.

Furthermore, publication bias might be in favor of positive results for the correlation between pain intensity and body composition measures, but after visual inspection of funnel plots and the result of the Egger's regression, no evidence for publication bias was found. Further, sensitivity analysis showed that removing a single study at a time from the present meta‐analysis had little effect on the pooled estimates, as they were not affected by small or low‐quality studies, and both positive as well as negative associations were reported from the included studies. In addition, research bias might be present for those studies, which were included after additional material was provided by authors, because it was not their primary intention to measure the association between pain intensity and overweight or obesity.

Finally, this review focused on the association between pain intensity and different body composition measures for overweight and obesity in adults suffering from CNLBP but did not investigate whether lean tissue mass was inversely related to pain intensity.

## 5 CONCLUSIONS

Based on the included set of studies in this systematic review and meta‐analysis, very low‐quality evidence for a positive, very weak association between pain intensity and body composition measures in adults with overweight and obesity suffering from CNLBP was found. Therefore, no clear interpretation can be drawn to corroborate the potential physiological and endocrine mechanisms of overweight or obesity (i.e., of adipose tissue) and their interaction with pain intensity. As those results are only attributed to selected study samples and based on mostly cross‐sectional studies of low quality, no causal conclusions can be drawn. Consequently, the evidence from the present review and meta‐analysis is not sufficient to justify clinical recommendations for the management of CNLBP. As BMI does not account for body fat distribution, other standardized measurements for overweight and obesity, such as body fat percentage, WC, visceral fat, and subcutaneous tissue fatness, are needed to evaluate their potential interaction with pain in future research. Randomized controlled trials are needed to investigate the causal effect of interventions on body composition and pain intensity in adults with overweight and obesity suffering from CNLBP.

## CONFLICT OF INTEREST STATEMENT

The authors declare that there is no conflict of interest.

## Supporting information


**Table S1.** Detailed search strategy for all databases.
**Table S2.** Quality appraisal of the included studies.
**Figure S1.** Funnel plot of the pooled studies investigating the correlation of pain intensity and BMI.
**Table S3.** Details about contacted authors.

## Data Availability

All data can be found in the manuscript. The search strategy, funnel plot, quality appraisal, and details about contacted authors can be found in the Supporting Information.
