# Supplementary material for: Association between pain intensity and body composition in adults with chronic non‐specific low back pain: A systematic review and meta‐analysis
Source: Obes Rev. 2024 Dec 2;26(4):e13875. doi: 10.1111/obr.13875 (PMC11884966; doi:10.1111/obr.13875)
Supplement: Supplementary file 1 — Table S1. Detailed search strategy for all databases. Table S2. Quality appraisal of the included studies. Figure S1. Funnel plot of the pooled studies investigating the correlation of pain intensity and BMI. Table S3. Details about contacted authors. [file OBR-26-e13875-s001.pdf]

Association Between Pain Intensity and Body Composition in Adults with Chronic Non-specific Low Back Pain: A Systematic Review and Meta-analysis

Authors:

Melanie Liechti [1,2,3], Massimo Menegon [1], Alexander Philipp Schurz [1,2,3,4], Jan Taeymans [1,2], Heiner Baur [1], Ron Clijsen [1,2,5,6], Anneleen Malfliet [3,7,8,9], Nathanael Lutz [1,2]

[1] School of Health Professions, Bern University of Applied Sciences, Bern, Switzerland

[2] Department of Movement and Sport Sciences, Faculty of Physical Education and Physiotherapy, Vrije Universiteit Brussel, Brussels, Belgium

[3] Pain in Motion Research Group (PAIN), Department of Physiotherapy, Human Physiology and Anatomy, Faculty of Physical Education and Physiotherapy, Vrije Universiteit Brussel, Laarbeeklaan 103, 1090 Brussels, Belgium

[4] Faculty of Medicine, University of Bern, Switzerland

[5] Rehabilitation and Exercise Science Laboratory RESLab, Department of Business Economics, Health, and Social Care, University of Applied Sciences and Arts of Southern Switzerland, Landquart/Manno, Switzerland; [6] International University of Applied Sciences THIM, Landquart, Switzerland

[7] Research Foundation – Flanders (FWO), Brussels, Belgium; [8] Chronic pain rehabilitation, Department of Physical Medicine and Physiotherapy, University Hospital Brussels, Belgium; [9] Pain in Motion International Research Consortium, www.paininmotion.be

Corresponding author: Melanie Liechti

Mailing address: Murtenstrasse 10, 3008 Bern, Switzerland

Telephone: +41 31 848 52 53

Email address: melanie.liechti@bfh.ch, Melanie.Nadia.Liechti@vub.be

Table S1 : Detailed search strategy for all databases

PubMed	("back pain"[MeSH Terms] OR "back pain"[Title/Abstract] OR "lumbar pain"[Title/Abstract] OR "lumbago"[Title/Abstract] OR "back ache"[Title/Abstract] OR "LBP"[Title/Abstract] OR "CLBP"[Title/Abstract] OR "NLBP"[Title/Abstract] OR "back disorders"[Title/Abstract] OR "chronic low back pain"[Title/Abstract])<br>AND<br>((("overweight"[MeSH Terms] OR "body weight"[MeSH Terms] OR "body constitution"[MeSH Terms] OR "adipose tissue"[MeSH Terms] OR "anthropometry"[MeSH Terms]) AND "overnutrition"[MeSH Terms]) OR "overweight"[Title/Abstract] OR "body mass index"[Title/Abstract] OR "bmi"[Title/Abstract] OR "adiposity"[Title/Abstract] OR "body composition"[Title/Abstract] OR "waist-hip ratio"[Title/Abstract] OR "total body fat mass"[Title/Abstract] OR "waist circumference"[Title/Abstract] OR "obesity"[Title/Abstract] OR "body weight"[Title/Abstract] OR "underweight"[Title/Abstract] OR "thinness"[Title/Abstract] OR "body fat distribution"[Title/Abstract] OR "body constitution"[Title/Abstract] OR "adipose tissue"[Title/Abstract] OR "anthropometry"[Title/Abstract] OR "overnutrition"[Title/Abstract])
CINAHL	MH (back pain OR low back pain) OR AB ("back pain" or "lumbar pain" or "lumbago" or "back ache" or "LBP" or "CLBP" or "NLBP" or "back disorders" or "CNSLBP" or "NSLBP" or "lumbar spine pain")<br>AND<br>MH (obesity or body weight or body constitution or adipose tissue or anthropometry or overnutrition or thinness or undernutrition or body mass index or adipose tissue distribution or body composition or waist-hip ratio or waist circumference) OR AB ("overweight" or "body mass index" or "adiposity" or "body composition" or "bmi" or "waist-hip ratio" or "total body fat mass" or "waist circumference" or "obesity" or "body weight" or "underweight" or "thinness" or "body fat distribution" or "body constitution" or "adipose tissue" or "anthropometry" or "overnutrition" or "high bmi" or "high body mass index" or "undernutrition")
Web of Science	TS=("back pain" or "lumbar pain" or "lumbago" or "back ache" or "LBP" or "CLBP" or "NLBP" or "back disorders" or "NSLBP" or "CNLBP")<br>AND<br>TS=("overweight" or "body mass index" or "adiposity" or "body composition" or "bmi" or "waist-hip ratio" or "total body fat mass" or "waist circumference" or "obesity" or "body weight" or "underweight" or "thinness" or "body fat distribution" or "body constitution" or "adipose tissue" or "anthropometry" or "overnutrition" or "body fat rate")

Cochrane library	MeSH descriptor: [Back Pain] explode all trees<br>("back pain" or "lumbar pain" or "lumbago" or "back ache" or "LBP" or "CLBP" or "back disorders" or "low back pain" or "non-specific chronic low back pain" or "non-specific chronic back pain" or "non-specific back pain" or "NSLBP"):ti,ab,kw<br>AND<br>MeSH descriptor: [Overweight] explode all trees<br>MeSH descriptor: [Body Weight] explode all trees<br>MeSH descriptor: [Body Constitution] explode all trees<br>MeSH descriptor: [Adipose Tissue] explode all trees<br>MeSH descriptor: [Anthropometry] explode all trees<br>MeSH descriptor: [Overnutrition] explode all trees<br>("overweight" or "body mass index" or "adiposity" or "body composition" or "bmi" or "waist-hip ratio" or "total body fat mass" or "waist circumference" or "obesity" or "body weight" or "underweight" or "thinness" or "body fat distribution" or "body constitution" or "anthropometry" or "overnutrition" or "body fat rate"):ti,ab,kw
Embase	('backache'/exp OR 'low back pain':ab,ti OR 'non-specific chronic back pain':ab,ti OR 'non-specific back pain':ab,ti OR 'cnlbp':ab,ti OR 'cnslbp':ab,ti OR 'nslbp':ab,ti OR 'back pain':ab,ti OR 'lumbar pain':ab,ti OR 'lumbago':ab,ti OR 'backache':ab,ti OR 'lbp':ab,ti OR 'clbp':ab,ti OR 'nlbp':ab,ti OR 'back disorders':ab,ti)<br>AND<br>('obesity'/de OR 'body weight'/de OR 'body constitution'/de OR 'adipose tissue'/de OR 'anthropometry'/de OR 'overnutrition'/de OR 'bmi'/de OR 'total body fat'/de OR 'body mass'/de OR 'underweight'/de OR 'body composition'/de OR 'waist hip ratio'/de OR 'waist circumference'/de OR 'body fat percentage'/de OR 'body fat mass'/de OR 'body fat distribution'/de OR 'body fat'/de OR 'body distribution'/de OR 'overweight':ab,ti OR 'body mass index':ab,ti OR 'bmi':ab,ti OR 'adiposity':ab,ti OR 'body composition':ab,ti OR 'waist hip ratio':ab,ti OR 'waist circumference':ab,ti OR 'obesity':ab,ti OR 'body weight':ab,ti OR 'underweight':ab,ti OR 'thinness':ab,ti OR 'body constitution':ab,ti OR 'adipose tissue':ab,ti OR 'anthropometry':ab,ti OR 'overnutrition':ab,ti OR 'body mass':ab,ti OR 'body fat':ab,ti OR 'body distribution':ab,ti) AND ([adult]/lim OR [aged]/lim OR [middle aged]/lim OR [very elderly]/lim OR [young adult]/lim)

Table S2: Quality appraisal of the included studies

Critical Appraisal Skills Programme (CASP) Checklist for Cohort Studies

**Study**<br>**Original Item Number**	1	2	3	4	5a	5b	6a	6b	9	10	11	12
Ansari (2022)*	Y	Y	C	C	C	N	NA	NA	Y	N	Y	Y
Barros dos Santos (2021)	Y	C	Y	Y	N	N	NA	NA	C	N	Y	N
Brooks (2016)	Y	Y	Y	Y	N	N	NA	NA	Y	C	C	Y
Chou (2016)	Y	Y	Y	Y	Y	Y	NA	NA	Y	Y	C	C
Ferrari (2019)	Y	Y	C	C	Y	Y	NA	NA	Y	N	Y	C
Gilmartin-Thomas (2020)	Y	Y	Y	Y	C	Y	NA	NA	Y	Y	Y	Y
Hussien (2019)	Y	Y	Y	Y	N	N	NA	NA	N	N	N	N
Iizuka (2015)	Y	Y	Y	Y	Y	Y	NA	NA	Y	Y	C	Y
Kastelic (2023)*	Y	Y	Y	Y	C	Y	NA	NA	Y	C	C	C
Kossi (2022)*	Y	Y	Y	Y	Y	Y	NA	NA	Y	Y	Y	Y
Ojoawo (2011)	Y	Y	Y	Y	N	N	NA	NA	C	N	Y	N
Prent (2023)*	Y	Y	Y	Y	Y	Y	NA	NA	Y	N	C	C
Sacomori (2010)*	C	C	C	C	N	N	NA	NA	N	N	Y	C
Sakulsriprasert (2021)	Y	Y	Y	C	N	C	NA	NA	Y	N	C	C
Shariat (2018)	Y	C	Y	N	C	N	NA	NA	C	N	Y	N
Siddiqui (2022)*	Y	Y	C	C	N	N	NA	NA	Y	Y	Y	Y
Sirbu (2023)	Y	Y	Y	Y	C	C	NA	NA	C	N	Y	C
Tavares (2020)	Y	C	C	Y	C	C	NA	NA	Y	N	N	N
Tsatsaraki (2023)*	Y	C	C	C	Y	Y	NA	NA	Y	N	Y	C
Urquhart (2011)	Y	C	Y	Y	Y	Y	NA	NA	Y	Y	C	C
Wood (2011)	Y	Y	C	N	N	N	NA	NA	N	C	C	N

Critical Appraisal Skills Programme (CASP) Checklist for Case Control Studies

**Original Item Number**	1	2	3	4	5	6a	6b	9	10	11
Carta (2023)*	Y	C	Y	Y	Y	Y	C	C	N	C

*Legend: *:* Additional calculations or raw data received from authors, *Abbreviations*: Y: Yes; C: Can’t tell; N: No; NA: Not applicable

Figure S1: Funnel plot of the pooled studies investigating the correlation of pain intensity and BMI

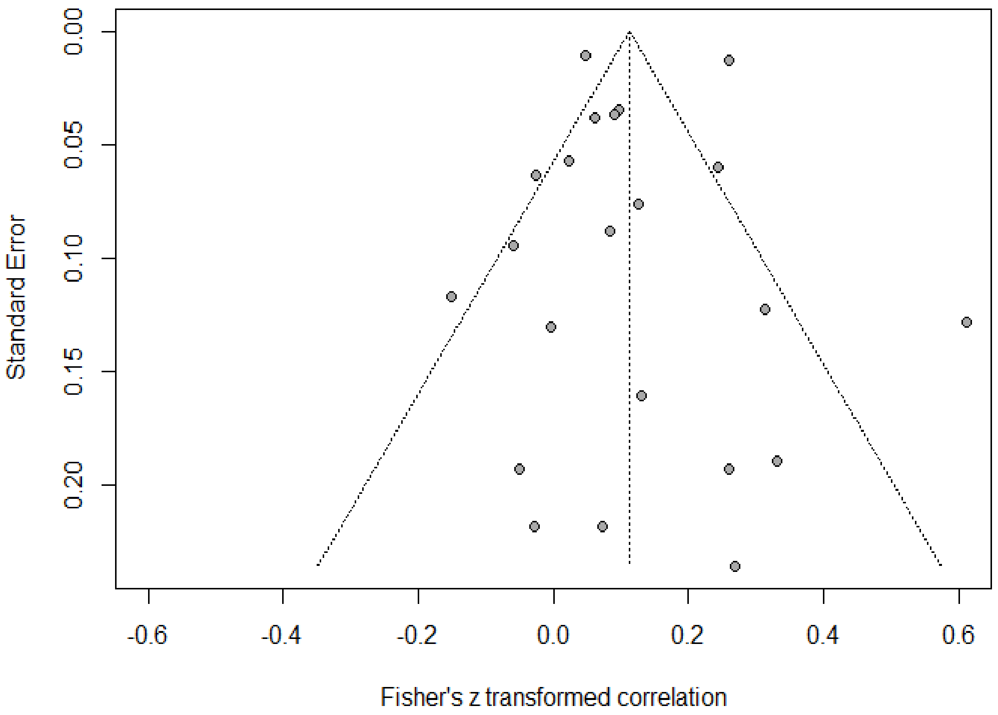

Table S3: Details about contacted authors

**Study of contacted authors**	**Author response**	**Inclusion/ Exclusion: Reason**
Higgins et al. 2020 [1]	Yes	Excluded: Different population
Shariat et al. 2018 [2]	Yes	Included: Authors confirmed that the type of pain was chronic
Jimenez-Trujillo et al. 2019 [3]	No	Excluded: No answer from authors
Brooks et al. 2016 [4]	No	Included: No answer from authors regarding analysis
Segar et al. 2016 [5]	Yes	Excluded: No data sharing possible, university authority agreement
Brady et al. 2019 [6]	No	Excluded: No answer from authors
Dave et al. 2019 [7]	No	Excluded: No answer from authors
Hashemi et al. 2016 [8]	No	Excluded: No answer from authors
Hussain et al. 2017 [9]	No	Excluded: No answer from authors
Okamoto et al. 2017 [10]	Yes	Excluded: No data sharing possible, institution does not allow the share data
Siddiqui et al. 2022 [11]	Yes	Included: Authors shared additional calculations
Urquhart et al. 2009 [12]	No	Excluded: No answer from authors
Ojoawo et al. 2011 [13]	Yes	Included: Authors confirmed CNLBP
Ewald et al. 2016 [14]	Yes	Excluded: No data sharing possible, no reason provided
Sharma et al. 2018 [15]	Yes	Excluded: Different population
Radhika Rao et al. 2011 [16]	Yes	Included: Raw data received
Sacomori et al. 2010 [17]	Yes	Included: Raw data received
Furtado et al. 2014 [18]	No	Excluded: No answer from authors
Marcus et al. 2004 [19]	No	Excluded: No answer from authors
Bolgen-Cimen et al. 2007 [20]	No	Excluded: No answer from authors
Parreira et al. 2017 [21]	No	Excluded: No answer from authors
Basem et al. 2021 [22]	No	Excluded: No answer from authors
Manchikanti et al. 2001 [23]	No	Excluded: No answer from authors
Sihawong et al. 2016 [24]	No	Excluded: No answer from authors
Yamakawa et al. 2004 [25]	Yes	Excluded: No data sharing possible, first author left and took password protected files with her
Ucar et al. 2021 [26]	Yes	Excluded: No response received anymore for data sharing
Hussain et al. 2016 [27]	Yes	Excluded: No data sharing possible, no ethics approval for sharing raw data
Larsson et al. 2016 [28]	No	Excluded: No answer from authors
Ansari et al. 2022 [29]	Yes	Included: Raw data received
Bansal et al. 2023 [30]	No	Excluded: No answer from authors
Kossi et al. 2022 [31]	Yes	Included: Raw data received
Prent et al. 2023 [32]	Yes	Included: Raw data received
Kastelic et al. 2023 [33]	Yes	Included: Raw data received
John et al. 2023 [34]	No	Excluded: No answer from authors
Tsatsaraki et al. 2023 [35]	Yes	Included: Raw data received
Carta et al. 2023 [36]	Yes	Included: Raw data received
Ho-A-Tham et al. 2022 [37]	No	Excluded: No answer from authors
Niu et al. 2024 [38]	No	Excluded: No answer from authors
Tavares et al. 2020 [39]	No	Included: No answer from authors regarding analysis
Sakai et al. 2023 [40]	No	Excluded: No answer from authors

1 Higgins DM, Buta E, Heapy AA*, et al.* The Relationship Between Body Mass Index and Pain Intensity Among Veterans with Musculoskeletal Disorders: Findings from the MSD Cohort Study. *Pain Med*. 2020; 21: 2563-72.
2 Shariat A, Cardoso JR, Cleland JA*, et al.* Prevalence rate of neck, shoulder and lower back pain in association with age, body mass index and gender among Malaysian office workers. *Work*. 2018; 60: 191-99.
3 Jimenez-Trujillo I, Lopez-de-Andres A, Del Barrio JL*, et al.* Gender Differences in the Prevalence and Characteristics of Pain in Spain: Report from a Population-Based Study. *Pain Med*. 2019; 20: 2349-59.
4 Brooks C, Siegler JC, Marshall PWM. Relative abdominal adiposity is associated with chronic low back pain: a preliminary explorative study. *BMC Public Health*. 2016; 16.
5 Segar AH, Urban JPG, Fairbank JCT, Judge A, Genodisc C. The Association Between Body Mass Index (BMI) and Back or Leg Pain in Patients With Spinal Conditions: Results from the Genodisc Study. *Spine (Phila Pa 1976)*. 2016; 41: E1237-E43.
6 Brady SRE, Urquhart DM, Hussain SM*, et al.* High baseline fat mass, but not lean tissue mass, is associated with high intensity low back pain and disability in community-based adults. *Arthritis Res Ther*. 2019; 21: 165.
7 Dave VR, Khanpara HJ, Shukla RP*, et al.* Risk factors of occupation related back pain and neck pain among patients attending tertiary care hospital, Ahmedabad, India. *J Prev Med Hyg*. 2019; 60: E419-E27.

8 Hashemi SM, Rohanifar R, Azarfarin R, Razavi SS, Momenzadeh S. A Comparison of the Sociodemographic and Clinical Characteristics of Patients Referring to a Pain Clinic with Subacute and Chronic Pain. *Anesth Pain Med*. 2016; 6: e39373.
9 Hussain SM, Urquhart DM, Wang Y*, et al.* Fat mass and fat distribution are associated with low back pain intensity and disability: results from a cohort study. *Arthritis Res Ther*. 2017; 19: 26.
10 Okamoto CS, Dunn AS, Green BN, Formolo LR, Chicoine D. Correlation of Body Composition and Low Back Pain Severity in a Cross-Section of US Veterans. *J Manipulative Physiol Ther*. 2017; 40: 358-64.
11 Siddiqui AS, Javed S, Abbasi S, Baig T, Afshan G. Association Between Low Back Pain and Body Mass Index in Pakistani Population: Analysis of the Software Bank Data. *Cureus*. 2022; 14: e23645.
12 Urquhart DM, Bell R, Cicuttini FM*, et al.* Low back pain and disability in community-based women: prevalence and associated factors. *Menopause*. 2009; 16: 24-9.
13 Ojoawo AO, Oloagun MOB, Bamiwoye SO. Relationship Between Pain Intensity and Anthropometric Indices in Women with low back pain - A Cross-Sectional Study. *j Phys Ther*. 2011; 3: 45-51.
14 Ewald SC, Hurwitz EL, Kizhakkeveettil A. The effect of obesity on treatment outcomes for low back pain. *Chiropr Man Therap*. 2016; 24: 48.
15 Sharma A, Shahi s, Begum R, Singh N. To Find Out the Correlation between Pain Disability and Quality of Life with Low back Pain in Housewives of Age Group 40–50 Years. *Indian Journal of Physiotherapy and Occupational Therapy - An International Journal*. 2018; 12.
16 Radhika Rao K. A study to determine the prevalence and knowledge of low back pain among students of selected nursing institutions in Udupi and Dakshina Kannada District, Karnataka. *International Journal of Nursing Education*. 2011; 3: 82-85.
17 Sacomori C, Fabiana Flores Sperandio AFM, Fernando Luiz Cardoso, Gilmar Moraes Santos. Self-reported low back pain in working women from seven rural communities in Concordia -SC. *Ter Man*. 2010; 8: 10-16.
18 Furtado RNV, Ribeiro LH, de Arruda Abdo B*, et al.* Nonspecific low back pain in young adults: Associated risk factors. *Revista Brasileira de Reumatologia (English Edition)*. 2014; 54: 371-77.
19 Marcus DA. Obesity and the Impact of Chronic Pain. *Clin J Pain*. 2004; 20: 186-91.
20 Bolgen-Cimen O, Arýncý-Ýncel N, Karabiber ME, C. Role of Obesity in Low Back Pain Related Disability. *West Indian Med J*. 2007; 56.
21 Parreira PCS, Maher CG, Ferreira ML*, et al.* A longitudinal study of the influence of comorbidities and lifestyle factors on low back pain in older men. *Pain*. 2017; 158: 1571-76.
22 Basem IJ, White RS, Chen SA*, et al.* The effect of obesity on pain severity and pain interference. *Pain Manag*. 2021; 11: 571-81.
23 Manchikanti L, Pampati V, Fellows B*, et al.* Characteristics of Chronic Low Back Pain in Patients in an Interventional Pain Management Setting: A Prospective Evaluation. *Pain Physician*. 2001; 4: 131-42.
24 Sihawong R, Sitthipornvorakul E, Paksaichol A, Janwantanakul P. Predictors for chronic neck and low back pain in office workers: a 1-year prospective cohort study. *J Occup Health*. 2016; 58: 16-24.
25 Yamakawa K, Tsai CK, Haig AJ, Miner JA, Harris MJ. Relationship between ambulation and obesity in older persons with and without low back pain. *Int J Obes Relat Metab Disord*. 2004; 28: 137-43.
26 Ucar I, Kararti C, Cuce I*, et al.* The relationship between muscle size, obesity, body fat ratio, pain and disability in individuals with and without nonspecific low back pain. *Clin Anat*. 2021; 34: 1201-07.
27 Hussain SM, Urquhart DM, Wang Y*, et al.* Associations between television viewing and physical activity and low back pain in community-based adults: A cohort study. *Medicine (Baltimore)*. 2016; 95: e3963.
28 Larsson C, Hansson EE, Sundquist K, Jakobsson U. Chronic pain in older adults: prevalence, incidence, and risk factors. *Scand J Rheumatol*. 2016; 46: 317-25.
29 Ansari S, Sharma S. Prevalence and risk factors of chronic low back pain in university athletes: a cross-sectional study. *Phys Sportsmed*. 2022; 51: 361-70.
30 Bansal D, Favas M, Bharti SK, Ghai B. Identifying Risk Factors and Health Concerns Associated With Chronic Low Back Pain Among Northern Indians: A Community-based Study. *Pain Physician*. 2023; 26.
31 Kossi O, Yamadjako D, Timmermans A*, et al.* Prevalence and biopsychosocial factors associated with chronic low back pain in urban and rural communities in Western Africa: a population-based door-to-door survey in Benin. *Eur Spine J*. 2022; 31: 2897-906.
32 Prent JM, van der Wurff P, Scholten-Peeters GGM. Lifestyle factors and psychological factors are associated with central pain processing in service members with persistent low-back pain: A cross-sectional exploratory study. *Medicine (Baltimore)*. 2023; 102: e36741.
33 Kastelic K, Sarabon N, Stanford T, Dumuid D, Pedisic Z. Are reallocations of time between physical activity, sedentary behaviour and sleep associated with low back pain? A compositional data analysis. *BMJ Open Sport Exerc Med*. 2023; 9: e001701.
34 John JN, Ugwu EC, Okezue OC*, et al.* Kinesiophobia and associated factors among patients with chronic non-specific low back pain. *Disabil Rehabil*. 2023; 45: 2651-59.
35 Tsatsaraki E, Bouloukaki I, Kontakis G, Vakis AF, Basta M. Associations between Combined Psychological and Lifestyle Factors with Pain Intensity and/or Disability in Patients with Chronic Low Back Pain: A Cross-Sectional Study. *Healthcare (Basel)*. 2023; 11.

36 Carta G, Costantini G, Garzonio S, Romano D. Investigation of the Relevant Factors in the Complexity of Chronic Low Back Pain Patients With a Physiotherapy Prescription: A Network Analysis Approach Comparing Chronic Pain-Free Individuals and Chronic Patients. *Am J Phys Med Rehabil*. 2023; 102: 571-76.

37 Ho-A-Tham N, Struyf N, Ting AKB*, et al.* Physical activity, fear avoidance beliefs and level of disability in a multi-ethnic female population with chronic low back pain in Suriname: A population-based study. *PLoS One*. 2022; 17: e0276974.

38 Niu S, Yang H, Gao J*, et al.* Correlation between sagittal parameters and disability of patients with nonspecific chronic low back pain: a cross-sectional study of 435 subjects. *Spine J*. 2024; 24: 634-43.

39 Tavares JMA, Rodacki ALF, Hoflinger F*, et al.* Physical Performance, Anthropometrics and Functional Characteristics Influence the Intensity of Nonspecific Chronic Low Back Pain in Military Police Officers. *Int J Environ Res Public Health*. 2020; 17.

40 Sakai Y, Watanabe T, Wakao N*, et al.* Skeletal Muscle and Fat Mass Reflect Chronic Pain in Older Adult. *Gerontol Geriatr Med*. 2023; 9: 23337214231190146.
